# How to achieve green development? A study on spatiotemporal differentiation and influence factors of green development efficiency in China

**DOI:** 10.1371/journal.pone.0291468

**Published:** 2024-01-25

**Authors:** Xia Zou, Yaping Xiao, Dalai Ma, Fengtai Zhang, Bitan An, Zuman Guo, Jiawei Zhang

**Affiliations:** 1 School of Management, Chongqing University of Technology, Chongqing, China; 2 Rural Revitalization and Regional High-quality Development Research Center, Chongqing University of Technology, Chongqing, China; Sichuan University, CHINA

## Abstract

For a long time, China ’s extensive economic development model has produced a large amount of emissions, which has brought indelible damage to the environment. Green development is of vital importance for China to achieve high-quality development, and it is the core of alleviating environmental problems and promoting sustainable development. How to achieve China ’s green development requires us to evaluate the level of green development in China ’s provinces and analyze the reasons. In this study, an evaluation index system including undesired output of green development efficiency is constructed, and then the Supe-SBM model is used to assess the green development efficiency of 30 Chinese provinces. This paper also discusses the spatial and temporal differences as well as the factors affecting green development efficiency of green development efficiency among provinces. The findings demonstrate: (1) The green development efficiency in the eastern region is the highest, followed by the western region, while the central region has the lowest, but they all show a downward trend. (2) The spatial characteristics of green development efficiency are remarkable, according to the Global Moran’s I index. However, the results of local spatial agglomeration demonstrate "small agglomeration and large dispersion," with the majority of provinces exhibiting L-L agglomeration. (3) Technological Progress, Opening Up, Urbanization Level are positively correlated with the green development efficiency. Industrial Structure, Financial Development, Energy Structure and green development efficiency are significantly negatively correlated, while Environmental Regulation shows no significant impact.

## 1. Introduction

China ’s economy has achieved vigorous growth by relying on high-input production methods in recent years. However, the extensive expansion mode also brought about critical issues for China, including severe resource shortages, severe environmental pollution, and ecosystem destruction. How to change the mode of economic development is particularly urgent. China’s 19th National Congress clearly promoted ecological civilization construction of China to the height of the "Millennium Plan" for national development, and the economic transformation mode with the green development at its core has gradually become an unavoidable option for China to break through resource and environmental limits and realize sustainable development. Green development is a new development model, which has the characteristics of high production, low consumption and low pollution compared with the extensive development model in the past. It reduces the ecological impact of economic output while maintaining normal economic development.

How to achieve green development in China? The key lies in evaluating the quality of China ’s green development. Green development efficiency (GDE) is a key indicator for evaluating the level of green development and has become an important academic research topic. Despite being examined from several angles, these studies nevertheless have certain drawbacks. The spatial pattern and dynamic changes in GDE are not sufficiently observed in these studies, which instead place greater emphasis on data calculation [1].

In reality, some studies indicated that China’s provinces have strong geographical, economic and social links, which creates a large spatial dependence on their GDE [2]. For example, regional pollution had a significant spillover effect, which can have an impact on a broader scale and even exceed provincial boundaries [3]. The first geographical law of Tobler [4] also stated that the objects in the space are interconnected among regions. The relationship between places is closer the closer the distance. China spans 9.6 million square kilometers, with significant regional disparities in economic growth and resource endowment. In light of this, it is critical to investigate the temporal and spatial differentiation of GDE among Chinese provinces to assist the government in developing regionally varied energy-saving and emission-reduction project.

Compared with previous studies, this study is innovative in the following three aspects: First, DEA method is mature in environmental efficiency research and has been widely used in GDE research, but there is a lack of GDE research using Super-SBM model. We use Super-SBM model to study the green development efficiency of China provinces to get more accurate results. Secondly, scholars mostly analyze the green development efficiency from a static perspective, with the focus on the calculation of green development efficiency. Few studies have revealed the temporal and spatial evolution characteristics of GDE among provinces in China. In this paper, Global Moran ’I index and local indication of spatial association (Lisa) map are used to investigate the spatial relationship and local spatial agglomeration characteristics of inter-provincial green development efficiency. Thirdly, although the influencing factors of green development efficiency have been fully analyzed, few scholars considered the spatial effect in their analysis. In this paper, the spatial econometric model is used to analyze the influencing factors of GDE, and the spatial correlation is considered. When the GDE has a high spatial correlation, the spatial econometric method can significantly improve model estimation accuracy when compared to the non-spatial econometric method.

The remainder of this paper is structured as follows: Section 2 organizes and compiles the pertinent literature; Methods, indicators, and data sources are introduced in Section 3; The measurement results, geographical economic regression findings, and temporal and spatial differentiation are covered in Section 4; The summary of the findings are outlined in Section 5 along with the suggestions to policy makers.

## 2. Literature review

Different from the traditional economic efficiency evaluation index, the GDE examines undesired output and factors in resource and environmental restrictions [5]. Reinhard et al. [6] and Shaik et al. [7] took pollutant variables as an input in the efficiency evaluation of handling pollutants as an undesirable output in the early stages. However, it violated the theoretical assumptions of strong input disposal and a restricted production possibility set. Simultaneously, the undesirable output was multiplied by -1 in Seiford and Zhu [8], and then converted the negative undesirable output to a positive number, which was subsequently included in the efficiency calculation.

How to reasonably evaluate the GDE is the emphasis of contemporary research [9]. Popular efficiency evaluation methods include stochastic frontier approach (SFA)and data envelopment analysis (DEA). Among them, SFA is a parameter estimation approach that not only requires the construction of a certain function form, but also can only cope with a single output problem, making evaluating the efficiency of multi-input and multi-output systems challenging [10]. As a result of SFA’s lack of flexibility in evaluating efficiency, few research employed the SFA method to assess the GDE. DEA, in contrast to SFA, is a nonparametric estimation approach that can handle problems with numerous inputs and does not call for the dimensionless treatment of indicators, making it a method with significant adaptability to complex economic systems. Charnes et al. [11] first put forward the CCR model with constant return on scale, which was used to measure the efficiency of decision-making units under the premise of constant return on scale. Later, Banker et al. [12] put forward the BCC model with variable return on scale, which was used to measure the pure technical efficiency and scale efficiency. Then, considering the slack of input or output, Tone [13] proposed a non-radial and non-oriented SBM model based on slack variables. As a result, many academics employed the DEA to assess the GDE from many perspectives. The SBM model was employed by Zhou et al. [14], Guo et al. [15] and Peng [16] to assess green total factor productivity in China’s cities. The GDE at the province level in China was calculated using the SBM model by Chen et al. [17] and Liu et al. [1]. However, the SBM model still shows some shortcomings. It cannot compare the cases with the same efficiency 1 [18]. This issue can be successfully resolved using the super-efficiency model put forth by Andersen and Petersen [19]. Compared with SBM, The GDE can be evaluated more precisely using the super-SBM model. Nevertheless, few researchers used this model to assess the GDE. We chose the Super-SBM model for the GDE evaluation of each province in China to increase the accuracy.

Many studies also concentrated on the influencing factors of GDE. Scholars analyzed environmental regulation [20], education spending [21], local government competition [22], green technology innovation [23], foreign direct investment [24], financial agglomeration [25], digital economy [26], economic complexity [27] and other factors on the GDE. However, the majority of these research carried out empirical study on the GDE influencing factors using Tobit, OLS, and Generalized Method of Moments (GMM) models. All of them employ the non-spatial econometric method. When the independent variables have considerable spatial connection, the spatial econometric method is more appropriate [28]. When investigating the factors that influence GDE, spatial characteristics should be considered [29].

Considering the findings of the preceding research, this paper develops a GDE evaluation index system that use Super-SBM to quantify the GDE of 30 provinces in China from 2000 to 2020. The entire evaluation index system includes indicators for labor, capital, energy, and water resources as inputs, GDP as desirable output, and industrial wastewater discharge, industrial sulfur dioxide emissions, and industrial smoke (powder) dust emissions as undesirable outputs. The research then explores the spatial connection of inter-provincial GDE using Moran’s I index and Lisa map, as well as the factors influencing GDE using a spatial econometric model.

## 3. Material and methods

### 3.1 Super-SBM

To scientifically and reasonably evaluate the GDE, the premise is to choose a suitable evaluation method. The DEA model was proposed for the first time by Charnes et al., (1978) [11]. Which is an effective way for evaluating the GDE at present [15]. Conventional DEA models, such as the CCR and BCC model, are both radial models. Due to the fact that these two models do not account for the slackness of input or output, there is a situation where the measured efficiency value is exaggerated. A slack-based, non-radial, and non-oriented SBM model was created by Tone [13] to address the measurement error brought on by the conventional radial model. Because it incorporates undesirable output in addition to slack input and output, this method is more acceptable and accurate in measuring efficiency.

Despite the fact that the SBM model offers numerous advantages in terms of measuring efficiency, it also has certain drawbacks. It is difficult to compare the efficiency of many DMUs that have same efficiency value of 1. Combining the idea of super-efficiency, Tone [30] created the Super-SBM model. The Super-SBM model can solve this problem, which can sort numerous DMUs more effectively, and it has more advantages in measuring the GDE.

The Super-SBM model’s operation premise is as follows: It is expected that there are n DMUs in a production set. A fixed number of production elements must be put into each DMU in the operation process and can bring a certain amount of output. Each DMU is thought to have invested *m* units of production factor *X*, and desirable output *Y* of *z*_1_ units and undesirable output *B* of *z*_2_ units are created, respectively. The *j*-th DMU to be estimated is *DMU*_*j*_. *λ* represents the weight. The equation of the Super-SBM model is as follows.


$$\begin{array}{l} \varphi^{*}=\min\frac{1-\frac{1}{m}\sum_{i=1}^{m}\;\frac{s_{i}^{x-}}{x_{ij}}}{1+\frac{1}{z_{1}+z_{2}}(\sum_{r=1}^{z1}\;\frac{s_{r}^{y+}}{y_{rj}}+\sum_{l=1}^{z2}\;\frac{s_{l}^{b-}}{b_{lj}})}\\ s_{.}t_{.}\;x_{ij}\geq \sum_{\begin{array}{l} i=1\\ i\neq j \end{array}}^{n}\;x_{ij}\lambda_{j}-s_{i}^{x-},i=1,\ldots ,m\\ \;y_{rj}\leq \sum_{\begin{array}{l} r=1\\ r\neq j \end{array}}^{n}\;y_{rj}\lambda_{j}+s_{r}^{y+},r=1,\ldots ,z_{1}\\ \;b_{lj}\geq \sum_{\begin{array}{l} l=1\\ l\neq j \end{array}}^{n}\;b_{lj}\lambda_{j}-s_{l}^{b-},l=1,\ldots ,z_{2}\\ \;\lambda \geq 0,s_{i}^{x-},s_{r}^{y+},s_{l}^{b-}\geq 0 \end{array}$$ (1)

In Formula (1), *φ* is the GDE calculated for Super-SBM model. *x*_*ij*_, *y*_*rj*_, *b*_*lj*_ are the *i*-th input, *r*-th desirable output and the *l*-th undesirable output of the *j*-th DMU respectively; $s_{i}^{x-}$ is the relaxation term of the *i*-th input. $s_{r}^{y+}$ is the relaxation term of the *r*-th desirable output, and $s_{l}^{b-}$ represents the relaxation term of the *l*-th undesirable output. When $s_{i}^{x-}=s_{r}^{y+}=s_{l}^{b-}=0$, it demonstrates that the relaxation terms of the inputs, the desired output, and the undesirable outputs are all equal to zero, and the GDE is optimal at this time. However, when $s_{i}^{x-}>0$、$s_{r}^{y+}>0$、$s_{l}^{b-}>0$, it means that the inputs and the undesirable outputs are redundant, the desirable output is insufficient. At this time, the GDE has not been optimized, and to achieve continual GDE improvement, the relaxation items of inputs and outputs must be adjusted.

### 3.2 Spatial panel model

#### 3.2.1 Global Moran’ I index

The spatial dependence of green development is very strong [31, 32]. It is very important to investigate the spatial correlation of GDE for understanding its spatial distribution law. At present, the Moran’s I index is widely used to measure spatial correlation. Moran’ I indexes are classified as global or local. The following is the Global Moran’ I index expression formula [33]:

$$Global\;Moran's\;I=\frac{\sum_{i=1}^{n}\sum_{j=1}^{n}W_{ij}(x_{i}-\bar{x})(x_{j}-\bar{x})}{G^{2}\sum_{i=1}^{n}\sum_{j=1}^{n}W_{ij}}$$ (2)

In Formula (2), $G^{2}=\frac{1}{n}\sum_{i=1}^{n}{\left(x_{i}-\bar{x}\right)}^{2}$. $\bar{x}=(\sum_{i}x_{i})/n$ represents the average of all the observation values in the study area. *n* denotes the number of areas to be researched. *x*_*i*_ is the observed value in *i*-th area, and *x*_*j*_ represents the observed value in *j*-th area. The spatial weight matrix is denoted by *W*_*ij*_, which is an adjacency matrix.

After calculating the Global Moran ’I index, its significance needs to be further verified to ensure its authenticity. Generally speaking, the significance of Global Moran ’I index is tested by Z-score normal distribution method. The following is its expression:

$$Zd=\frac{\left[Global\;Moran's\;I-E(Global\;Moran's\;I)\right]}{\sqrt{VAR(Global\;Moran's\;I)}}$$ (3)

Where, $E_{n}(Moran's\;I)=-\frac{1}{n-1}$ represents the expected value of *Moran*’*s I*. $Var(Moran's\;I)=\frac{n^{2}w_{1}+nw_{2}+3w_{0}^{2}}{w_{0}^{2}(n^{2}-1)}-E_{n}^{2}(Moran's\;I)$ represents the variance of the *Moran*’*s I*. *w*_*i*_ denotes the sum of all spatial matrices’ values in *i*-th rows. *w*_∙*i*_ denotes the sum of all spatial matrices’ values in *i*-th columns. If the Z value of Global Moran’ I is 10%, 5%, or 1% significant, it implies the existence of Global Moran’ I and that the observed values in the studied area have significant spatial association.

#### 3.2.2 Local Moran’ I index

The Global Moran’ I index cannot account for the local spatial distribution of regional observations, so, the Local Moran’ I index must be introduced to investigate the local spatial correlation further. The spatial features of the observed values can be reflected by the Lisa map after calculating the Local Moran’ I index. The following is the Local Moran’ I index expression formula [34]:

$$Local\;Moran's\;I=S_{i}\sum_{j=1}^{n}w_{ij}S_{j}$$ (4)

*S*_*i*_ denotes the standardization of observation values in *i*-th area. *S*_*j*_ denotes the standardization of observation values in *j*-th area. *w*_*ij*_ denotes the standardization of spatial weight matrix rows.

#### 3.2.3 Spatial econometric model

What factors affect the inter-provincial GDE in China? This is the important goal of this paper. The traditional panel model based on ordinary least square method often ignores the possible spatial correlation of regional GDE when estimating [18]. To accurately examine the factors affecting GDE, the spatial econometric model considering spatial factors needs to be established. The spatial autoregressive model (SAR) and the spatial error model (SEM) are two classifications of traditional spatial econometric model. It is assumed that there are *n* influencing factors affecting the GDE, which is represented by the set *X* of influencing factors. The spatial econometric model of the GDE influencing variables is constructed by combining the two fundamental models of SAR and SEM. GDE is used as the dependent variable, and each influencing variable is treated as the independent variable. The following is the specific expression [35]:

$$\begin{array}{l} GDE_{i,t}=\alpha_{i}+\phi_{t}+\beta X_{i,t}+\delta \sum_{j}W_{ij}*(GDE_{i,t})+\mu_{i,t}\\ \mu_{i,t}=\lambda \sum_{j}W_{ij}*\mu_{j,t}+\varepsilon_{i,t} \end{array}$$ (5)

Eq (5) is the most basic general spatial fixed effect model constructed in this paper. *δ* and *λ* represent the spatial autoregressive and spatial error coefficients, respectively. If *δ* = 0, the model is transformed into the *SEM*; if *λ* = 0, the model is transformed into the *SAR*. The spatial fixed effect and the time fixed effect coefficients are represented by *α*_*i*_ and *ϕ*_*t*_, respectively. *X*_*i*,*t*_ denotes the set of *n* influencing factors of region *i* in year *t*. *μi*_,*t*_ denotes the random error term.

### 3.3 Indicators construction

#### 3.3.1 Input-output variables for measuring GDE

In essence, the GDE measures how effectively ecology, the environment, and resources are used overall with the aim of achieving efficiency, sustainability, and harmony [18]. This study defines GDE as a green total factor productivity that includes economy, resources, ecology, and environment [1, 36]. In order to accurately assess GDE, we develop an input-output index system [37] (Table 1). Among them, labor, capital, energy, and water resources are the four input indicators. GDP is the desired output. The undesirable output is industrial pollutants including wastewater discharge, sulfur dioxide discharge, and smoke (powder) dust emissions.

**Table 1:** The description of input-output indicators for GDE.

	Variables	Indicator description	Unit
**Input indicators**	Labor	Number of employed people of each region	Ten thousand people
	Capital	Real capital stock of each region	100 million yuan
	Energy	Energy consumption of each region	10,000 tons of standard coal
	Water resources	Total water supply of each region	100 million cubic meters
**Output indicators**	Desirable output	Real GDP of each region	Hundred million yuan
	Undesirable output	Industrial wastewater discharge of each region	Ten thousand tons
		Industrial sulfur dioxide emissions of each region	Ten thousand tons
		Industrial smoke (powder) dust emissions of each region	Ten thousand tons

1. **Labor**: Not only is labor a crucial component in achieving economic progress, but it also forms the foundation of sustainable development. It is denoted by the number of employed people of each region in this study [38].
2. **Capital**: Capital is essential for achieving economic progress, and it is also a part of financial investment in total factor productivity. This paper uses capital stock to express capital, The capital stock is calculated with the "perpetual inventory method," which Goldsmith [39] first proposed. The expression formula is $K_{it}=I_{it}+(1-\delta )K_{it-1}$. Among them, the capital stock of region *i* in year t is represented by *K*_*it*_. *K*_*i*(*t*−1)_ is the capital stock of region *i* in year (*t*−1). *I*_*t*_ is the fixed capital formation in *i*-th region in *t*-th period. And *δ* is the capital depreciation rate in *i*-th region in *t*-th period, the value here is 10.96%. Furthermore, we reduce the nominal capital stock of each area across time to the actual capital stock in the base period of 2000 to prevent the influence of pricing considerations according to Li et al. [40].
3. **Energy**: Energy is inextricably linked to economic and social production, and it is more than just the engine of economic growth, also the primary determinant of whether it can develop greenly. Based on the research results of Zhuo and Deng [41], the energy input in this study is expressed as the regional energy consumption.
4. **Water resources**: China has a sizable population, which is not only a huge water user, but also a country with poor water resources per capita. Considering the serious water resource constraints in China, some scholars take water resources as an input factor to measure efficiency [27, 42]. Therefore, the total water supply of each region over the years is used as the water resources input in this paper.
5. **Desirable output**: Like Pan et al. [43] and Feng et al. [44], the GDP of each region is used in this paper to indicate the desired output. Similarly, Similarly, the GDP price index is used to calculate the real GDP to eliminate the false high influence caused by price factors, with 2000 as the base period.
6. **Undesirable output**: Undesirable output refers to all kinds of pollutants and emissions that harm the environment that are produced by various factors inputs. Currently, air and water pollution are becoming more prevalent in China, and they mainly come from industrial pollution. Inspired by Guo et al. [15], this paper chooses industrial wastewater discharge, industrial sulfur dioxide discharge and industrial smoke (powder) dust emissions to measure the undesirable output.

#### 3.3.2 Factors influencing GDE

Investigating the GDE’s impacting variables can help to illuminate the motivations for China’s green development, thus providing important basis for policy recommendations. Yu et al. [29] classified the variables affecting China ’s inter-provincial GDE into economic, technical, resource and policy aspects. Yang et al. [45] investigated the impact of economic development level, technical progress, opening up, industrial structure, population density, and local government urban agglomerations on GDE.

The influencing factors of GDE are attributed to seven factors in this paper: Industrial Structure (IS), Technological Progress (TP), Opening Up (OU), Financial Development (FD), Environmental Regulation (ER), Energy Structure (ES), Urbanization Level (UL). Table 2 contains a list of the variables’ descriptions. The variables influencing GDE are explained by the following.

**Table 2:** The description of variables influencing GDE.

Variables	Variables Definition	Units
Industrial Structure (IS)	Secondary industry output value /GDP	%
Technological Progress (TP)	R&D value / GDP	%
Opening Up (OU)	Real FDI/ GDP	%
Financial Development (FG)	Sum of deposit and loan balances /GDP	%
Environmental Regulation (ER)	Industrial pollution control investment /Industrial added value	%
Energy Structure (ES)	Coal consumption/total energy consumption	%
Urbanization Level (UL)	Urban resident population /total population	%

1. **Industrial Structure (IS)**: It is measured by the percentage of primary, secondary, and tertiary industries in the national economy, which is the foundation and core of the transition of development mode, as well as an important supporting condition for improving GDE. Previous research demonstrated that the secondary industry is the main driver of energy use and environmental harm [46]. This study expresses the variable using the regional secondary industry to GDP. We initially think that industrial structure variable’s coefficient is negative.
2. **Technological Progress (**TP**)**: The improvement of production technology can be encouraged by technological progress, thereby saving the input in the production process and shortening the production cycle of products. It can also encourage businesses to use and develop renewable energy, progressively using less traditional fossil fuels and lowering pollutant emissions. It may be claimed that technology progress is the main factor driving changes in firm production modes, as well as the basic way of effectively realizing enterprise energy savings and emission reduction [47]. The ratio of R&D value to GDP is taken as a substitute indicator for technological progress in this paper, and this variable’s coefficient is preliminarily determined to be positive.
3. **Opening Up (OU)**: Generally speaking, foreign direct investment (FDI) is the main indicator to measure the level of a country ’s opening up. Previous studies showed that the ecological environment of the host nation is impacted by FDI in two separate ways. One is the "pollution paradise" hypothesis. Some scholars believed that with the improvement of opening up, the efforts to attract foreign investment made by the host nation continue to increase. Polluting industries will be transferred from developed countries to developing countries, severely harming the host country’s environment [48]. The other is the "pollution halo" hypothesis, which maintains that the entry of FDI by the host country has a positive effect on the spread of technology [49]. FDI can bring more cutting-edge technology, more skilled talents and advanced equipment, which can promote economic development, environmental governance and technological progress. The ratio of the real FDI to GDP in each region is used to denote the opening up after converting dollars into RMB in this paper. We preliminarily presume that there is some uncertainty between opening up and GDE.
4. **Financial Development (FD)**: Previous research demonstrated a clear "double effect" between financial development and the growth of the green economy [50]. First, the government can formulate financial policies to guide financial institutions to lend money to green projects that are conducive to environmental protection [51]. Additionally, businesses can use loan funds to modernize equipment and implement more environmentally friendly production techniques to lower pollutant emissions [52]. Besides, more funding sources for firms with high pollution are provided by financial development, which raises energy use and emissions of pollutants [53]. The ratio of sum of deposit and loan balances to GDP is used to represent financial development. In view of its influence on the GDE is uncertain, so the results of variable coefficient need to be further tested.
5. **Environmental Regulation (ER)**: Environmental regulation is an essential instrument for preventing businesses from releasing different types of environmental contaminants. However, environmental regulations generally have a "double-edged sword" effect on local energy saving and emission reduction, meaning that "forced emission reduction" and "green paradox" coexist. The view of "forced emission reduction" holds that businesses may be compelled by environmental regulations to conduct research on green environmental protection technologies. This will not only help businesses advance their production technology but will also encourage them to use cleaner energy sources more frequently [54]. The "Porter Hypothesis" also makes the argument that businesses can save energy by complying with tight and appropriate environmental regulations and reduce industrial pollutants. The view of "green paradox" holds that the production cost of enterprises will rise as a result of increased laws and regulations, which will have a negative influence on the GDE by driving up resource development and consumption [55]. The proportion of industrial pollution control investment to industrial added value is used as an alternative variable of environmental regulation in this study, and the coefficient of it is regarded as tentatively unknown.
6. **Energy Structure (ES)**: Energy is divided into clean energy and polluting energy. Among them, fossil energy represented by coal, oil is a typical polluting energy. The region’s environmental quality will decline as a result of the use of use of these polluting energy, which will produce a lot of pollutants. In contrast, renewable energy represented by hydropower, solar energy and wind energy is a typical clean energy. The usage of clean energy has undoubtedly contributed favorably to the improvement of environmental standards. As China is a big country of coal production and consumption, the energy structure is depicted in this study with the proportion of coal consumption in total energy consumption in each region. We initially assume that the energy structure coefficient is negative.
7. **Urbanization Level (UL)**: Urbanization has a wide-ranging effect on green development. First, it has aided the growth of industrialization by advancing urbanization continuously. The city, as the primary carrier of industrialization, will unavoidably produce more industrial ’three wastes’ emissions and impair the environment due to its use of fossil fuels [56]. In addition, urbanization will result in the "agglomeration effect" of production factors, as more labor, technology, and capital will congregate in cities, boosting the development of urban production efficiency. With the gradual deepening of urbanization, urban infrastructure will be improved continuously, and citizens’ knowledge of environmental protection will rise. These factors will support economic growth and be crucial in addressing energy and environmental issues. The proportion of urban resident population to the total population serves as a proxy for the urbanization level in this study, and it is assumed that the coefficient of this variable is uncertain.

### 3.4 Data sources

The research object of this paper is 30 provinces in China, and the research span is 2000–2020. Tibet, Hong Kong, Macao, and Taiwan in China cannot be included in the study because their data are seriously lacking. All variables in this research are based on data from the China Statistical Year (2001–2021), China Statistical Year on Science and Technology (2001–2021), China Energy Statistical Yearbook (2001–2021) and China Yearbook on Environment (2001–2021). In addition, the data of a few variables come from Chinese provincial statistical yearbooks.

## 4. Results and discussion

### 4.1 Spatiotemporal characteristics of GDE

**Fig 1:**
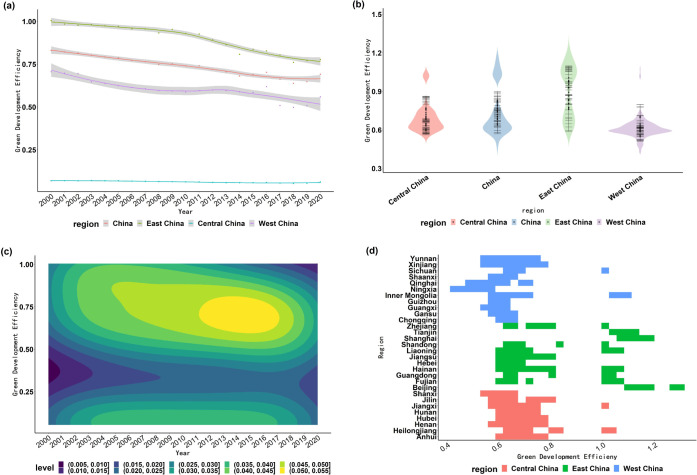
National, regional, and provincial GDE. Note: (a) shows the curve plot of average GDE for China and the three regions. (b) shows the violin plot of average GDE for China and the three regions. (c) shows the two-dimensional density plot of average GDE for China. (d) shows the distribution of provincial GDE.

China’s provinces are divided into three regions according to their geographical location and level of economic development, namely, the eastern region, the central region and the western region. Fig 1 analyzes the GDE from the perspectives of the nation, the region, and the province, and discovers the following characteristics. From the national perspective (Fig 1(C)), the GDE is primarily concentrated in the middle level, with an efficiency value greater than 0.5, then comes the low level and the high level. The middle-low level region has the lowest density during the study period, with a density of 0.25–0.5. In general, GDE in China has been largely below the middle level in recent years, so much room for improvement in GDE. In particular, China’s GDE still has the trend of gathering area moving down, and steps must be taken to prevent this trend from worsening in time.

At the regional perspective (Fig 1(A) and 1(B)), the average GDE of the three regions is in a fluctuating downward trend, and always shows that eastern region > western region > central region. Among them, the average GDE in the eastern region is even higher than the national level, but the downward trend is the most significant during the study period. While the average GDE in the central and western regions is lower than the national level, especially in the central region. It can be seen that since 2000, the GDE in the nation and the three major regions has shown a decreasing trend.

From the provincial perspective (Fig 1(C) and 1(D)), only Tianjin, Shanghai, and Beijing reach the forefront of production and achieve the best GDE, whereas other provinces do not. The GDE in provinces such as Guangdong and Fujian is not optimal, but it is close to the efficiency frontier level. However, the GDE of Gansu, Ningxia, Shanxi, Shaanxi, Sichuan, Chongqing, Guizhou, Hunan, Guangxi and other provinces in the central and western regions is not ideal, which needs to be improved to a great extent in the future. In a word, the GDE of different provinces is quite different. On the whole, most eastern coastal provinces have relatively high GDE, while most inland provinces have relatively low GDE.

**Fig 2:**
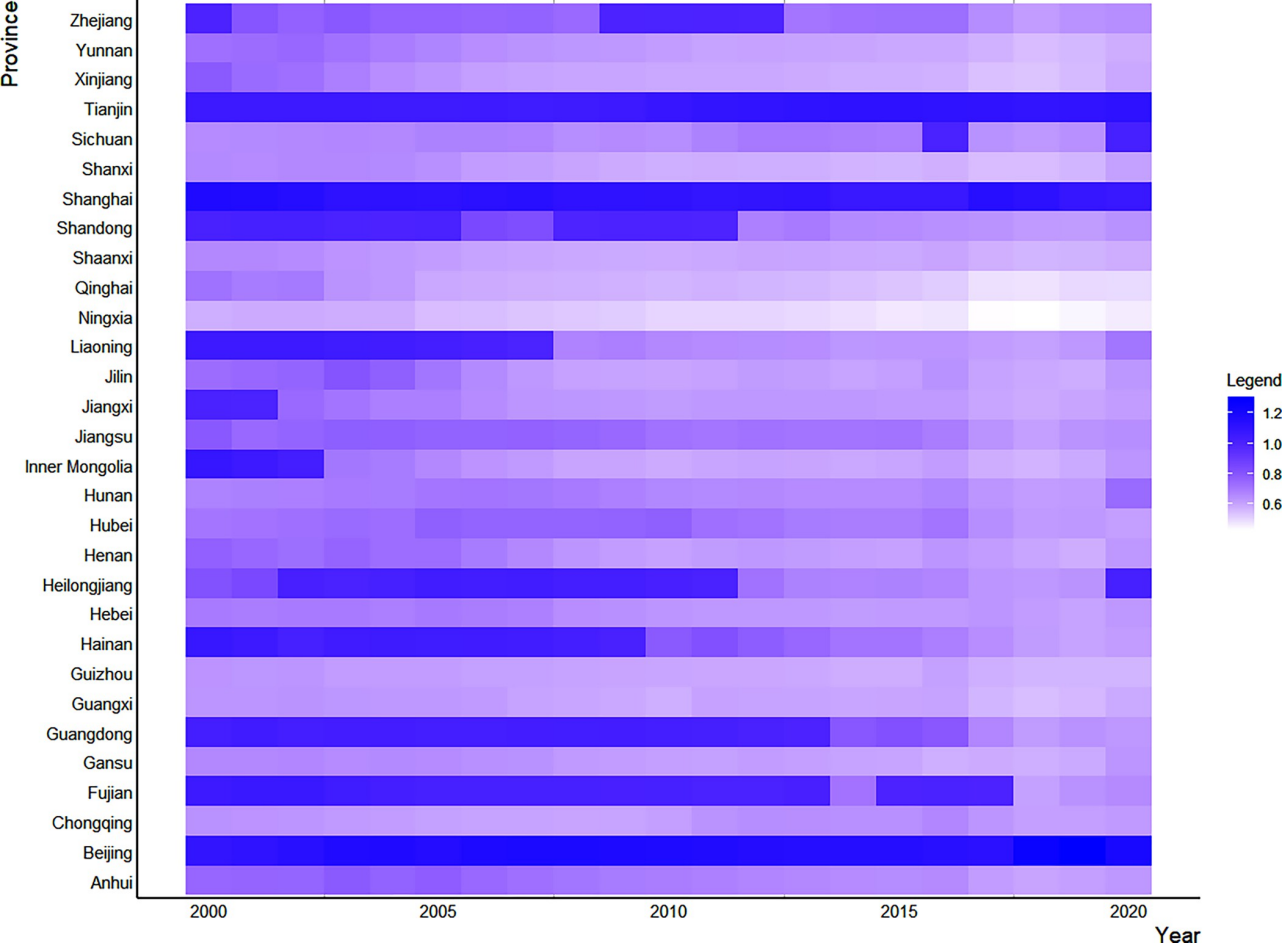
Spatial and temporal distribution of GDE.

We have drawn the spatiotemporal distribution figure of GDE in various provinces to more intuitively display the temporal and spatial distribution of China, as shown in Fig 2.

In terms of provinces, the GDE in China shows great spatial differences, and changes with time. In this study, we divide the GDE in China into 5 levels from low to high, represented by different colors. In 2000, Inner Mongolia, Liaoning, Beijing, Tianjin, Shanghai and Fujian are at the first level, and their GDE reaches the forefront of production. Along with time, great changes take place in the provinces at the first level. Only Beijing, Tianjin and Shanghai have been at the first level in GDE. The GDE in Inner Mongolia, Liaoning and Fujian has decreased. It may be because these provinces relied heavily on energy to achieve quick economic development early period. But the negative environmental impact of this high-input and high-emission production model has gradually become prominent over time. Heilongjiang, Guangdong and Sichuan have reached the first level in some years, and their GDE has been effectively improved. This shows that the green development strategy adopted by these provinces in recent years has achieved good results. Provinces with low GDE should actively learn green development experience from them.

In 2000, Gansu, Ningxia, Shanxi, Shaanxi, Sichuan, Chongqing, Guizhou, Hunan and Guangxi are in the fifth level. Some provinces have improved their GDE over time, while Ningxia’s GDE is still in the fifth level. Guizhou and Guangxi increase their GDE to the fourth level, while Hunan, Chongqing, and Shaanxi increase to the third level, although their GDE values remain low. Only Sichuan has achieved a significant improvement in GDE, which is at the second level in 2016 and 2020. Other provinces’ GDE varies substantially. Shandong, Zhejiang, Jiangxi, Hubei, and Henan are in the second level in some years, but drop to the fourth and fifth levels in others, indicating that there is much space for improvement in their GDE.

Regionally, the GDE in the three major regions of China is quite different. The majority of the provinces with high GDE are in eastern China, including Beijing, Tianjin, Shanghai, Guangdong, and Fujian. The middle and western areas are where the majority of the provinces with low GDE. So, the central and western regions are critical areas of energy conservation and emission reduction that require special attention. In addition, China’s provinces are more engaged in internal cooperation, but less in cross-regional cooperation, which further leads to differences by region of the GDE in China.

### 4.2 Spatial correlation analysis of the GDE

#### 4.2.1 Global spatial correlation test results

In this paper, the Global Moran’s I index of the GDE is calculated by Arcgis10.2, as shown in Table 3. Table 3 demonstrates that all the Global Moran’s I index is positive, and all the other years pass the test of 10%, 5% or 1% significance level except for 2020. The results show that there is a significant positive spatial correlation in GDE. It also implies that spatial agglomeration is a common phenomenon of China’s GDE, not randomness. In other words, the GDE of neighboring provinces has a strong imitation effect.

**Table 3:** Global Moran’s I index of GDE.

Year	Moran’s I index	Expectation index	Variance	Z score	P value
2000	0.2773	-0.0345	0.0156	2.4950	0.0126
2001	0.1994	-0.0345	0.0156	1.8754	0.0607
2002	0.20471	-0.0345	0.0155	1.9197	0.0549
2003	0.2889	-0.0345	0.0154	2.6053	0.0092
2004	0.2572	-0.0345	0.0154	2.3513	0.0187
2005	0.2401	-0.0345	0.0154	2.21081	0.0271
2006	0.2300	-0.0345	0.0153	2.1406	0.0323
2007	0.2188	-0.0345	0.0152	2.0517	0.0402
2008	0.2473	-0.0345	0.0152	2.2853	0.0223
2009	0.3140	-0.0345	0.0154	2.8103	0.0050
2010	0.3196	-0.0345	0.0152	2.8684	0.0041
2011	0.3182	-0.0345	0.0152	2.8579	0.0043
2012	0.4392	-0.0345	0.0147	3.9081	0.0001
2013	0.3416	-0.0345	0.0143	3.1495	0.0016
2014	0.4116	-0.0345	0.0130	3.9074	0.0001
2015	0.3834	-0.0345	0.0139	3.5486	0.0004
2016	0.2152	-0.0345	0.0144	2.0824	0.0373
2017	0.3141	-0.0345	0.0135	2.9976	0.0027
2018	0.3686	-0.0345	0.0120	3.6788	0.0002
2019	0.3766	-0.0345	0.0116	3.8072	0.0001
2020	0.1367	-0.0345	0.0141	1.4409	0.1496

#### 4.2.2 Local spatial correlation test results

**Fig 3:**
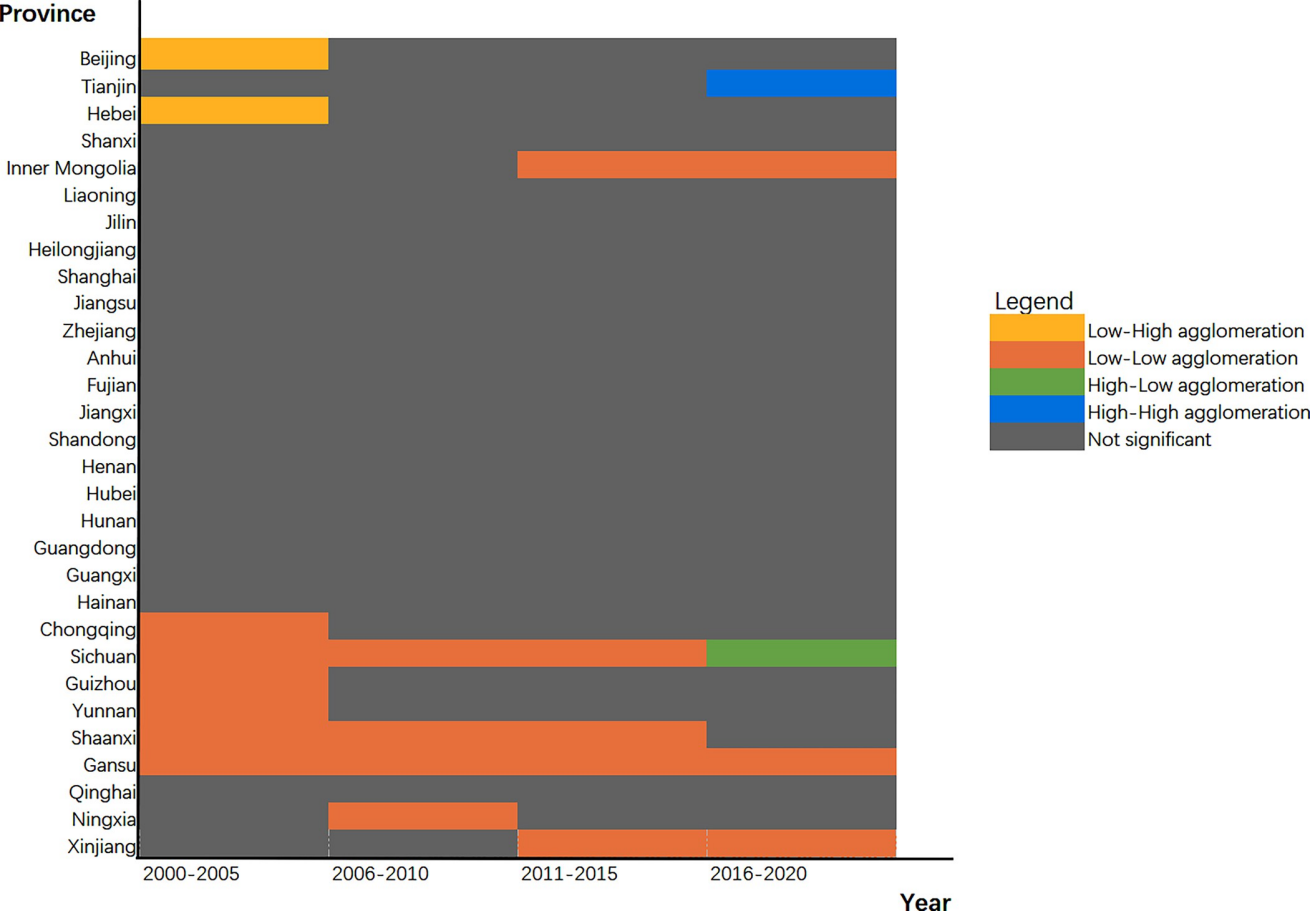
The spatial correlation of provincial GDE.

The Lisa map is created to better examine the local spatial agglomeration features of inter-provincial GDE in China, and on this basis, the agglomeration features of GDE in each province are further analyzed, as shown in Fig 3.

In general, the GDE from 2000 to 2020 exhibits "small agglomeration and large dispersion," primarily L-L agglomeration. GDE is low in both of these provinces and in their neighboring provinces. This demonstrates that China’s GDE has not been effectively improved, which gives a warning to China’s green development. At present, China is still dominated by traditional production methods. This intensive production has caused a lot of pollutant emissions and significant environmental damage.

Specifically, the provinces with L-L concentration from 2000 to 2005 include Gansu, Shaanxi, Sichuan, Chongqing, Yunnan and Guizhou. From 2006 to 2010, Ningxia is added to the provinces with L-L agglomeration, while Chongqing, Yunnan and Guizhou are reduced. The situation of L-L agglomeration has improved. In 2011–2015, Xinjiang and Inner Mongolia are added to the provinces with L-L agglomeration, while Ningxia is reduced. The scope of L-L agglomeration is expanding. In 2016–2020, the provinces with L-L concentration decrease in Shaanxi and Sichuan. The scope of L-L agglomeration is shrinking. To sum up, the provinces with L-L concentration show strong volatility during the study period. Although the geographic range of L-L agglomeration has narrowed in some years, the situation of L-L agglomeration has not fundamentally changed. Regionally, provinces having a high concentration of L-L are primarily found in the central and western China. This demonstrates that the western provinces have stronger spatial dependence, and the provinces with low GDE will affect the neighboring provinces, making the neighboring provinces also show low GDE. China should concentrate its efforts in the central and west in the future to prevent the L-L gathering area from spreading. From the perspective of provinces, most of the L-L agglomeration’s provinces are underdeveloped inland areas in China. These provinces have no strong economic input to support their technological upgrading and industrial transformation, and their geographical conditions are backward, so it is easier to show L-L agglomeration with low GDE.

### 4.3 Analysis of influencing factors

#### 4.3.1 Ordinary model and residual spatial correlation test results

Based on Formula (5), the regression model of influencing factors of GDE is preliminarily simulated in this paper. Table 4 displays the specific outcomes, which also gives the estimation coefficient of each variable and the spatial correlation test results of model residual term. Furthermore, we can determine which no fixed effect model is optimal comparing the estimation results of the no fixed effect model, spatial fixed effect model, time fixed effect model, and two-way fixed effect model for mutual comparison.

**Table 4:** Estimation and different panel data models.

Variables	No fixed effect	Spatial fixed effect	Time fixed effect	Two-way fixed effect
Industrial Structure (IS)	-0.1404* (-1.6786)	0.0586 (0.7469)	-0.4132*** (-5.9130)	-0.3288 (-3.4162)
Technological Progress (TP)	4.6306*** (6.5497)	-3.5248*** (-2.9912)	2.0427*** (3.5644)	-1.5600 (-1.3580)
Opening Up (OU)	2.7994*** (9.9786)	0.4607** (1.9326)	0.5899** (2.3899)	0.1351 (0.5929)
Financial Development (FG)	-0.0975*** (-5.2401)	0.0131 (0.7012)	-0.0764*** (-4.9498)	0.0204 (0.9949)
Environmental Regulation (ER)	5.3381*** (3.3926)	-0.1815 (-0.1356)	-2.1688* (-1.5871)	-0.9012 (-0.6606)
Energy Structure (ES)	-0.3833*** (-9.3210)	-0.1544** (-2.9822)	-0.2373*** (-7.1816)	-0.2374*** (-4.6570)
Urbanization Level (UL)	0.1764*** (2.9200)	-0.5453*** (-8.1263)	0.7776*** (13.8405)	0.5001*** (3.6504)
*R*−*squared*	0.5423	0.2785	0.6954	0.0567
*DW*	1.4764	1.8541	1.8921	2.1199
*LM*−*lag*	94.2286***	58.3896***	15.5164***	13.7489***
*Robust LM*−*lag*	19.0839***	81.7111***	13.0383***	135.7749
*LM*−*err*	75.2948***	33.3289***	4.7549**	3.3663*
*Robust LM*−*err*	0.1502	56.6505***	2.2769	125.3923***

Note: The values in () are T-test values, and *, * * and * * represent the significance levels of 10%, 5% and 1% respectively. Model estimation and spatial autocorrelation testing are conducted using Matlab7.12.

According to the results in Table 4, the *R−squared* of the time fixed effect model is 0.6954, which is clearly superior than the no fixed effect model, spatial fixed effect model and two-way fixed effect model. In addition, the *DW* of the time fixed effect model is 1.8921, which is smaller than that of the two-way fixed effect model, but larger than that of the no fixed effect model and the spatial fixed effect model. From the above results, we find that the time-fixed effect model is the best, so we use the outcomes of this model to analyze the explanatory significance of each variable coefficient.

The lower part of Table 4 displays the test results for the spatial correlation of model residuals. By comparing the size and significance of Lagrange Multiplier statistics (LM), we can choose whether to adopt the SAR or the SEM model. The *LM*−*lag* of time fixed effect model is 15.5164, and it is significant at 1% level, the *LM*−*err* of time-fixed effect model is 4.7549, and it is significant at 5% level. This finding demonstrates that the ordinary model’s residual term exhibits strong spatial correlation, and the estimation result using the common model may be biased. To increase the accuracy of the estimation findings, we use the spatial econometric model for re-regression. In addition, the *LM*−*lag* is manifestly bigger than the *LM*−*err*, so the SAR model’s estimation result is superior to that of the SEM model.

#### 4.3.2. Analysis of influencing factors results

In view of the obvious spatial correlation of the residual term of the ordinary model, we again regress the model (5) using the spatial econometric model. The SAR model and SEM model’s outcomes are shown in Table 5. The W*dep.var. of the SAR model is 0.1550, which it is significant at 1% level. The *spat*.*aut* of SEM model also passes the test of 1% significance level. This demonstrates how important it is to use a spatial econometric model. In addition, the spatial econometric model’s *R−squared* is higher than that of the ordinary model, and the values of the t-test some variables are improved, which fully demonstrates that the spatial econometric model’s estimation result is superior to the ordinary model. Furthermore, because the SAR model has a greater *R−squared* than the SEM model, the SAR model has a better fitting degree. In view of this, we use the SAR model’s estimation outcomes to discuss the influence of various factors on the GDE.

**Table 5:** Estimation of the time fixed effect spatial economic model.

Variables	SAR	SEM
Industrial Structure (IS)	-0.3727*** (-5.4005)	0.4010*** (-5.8563)
Technological Progress(TP)	2.1249*** (3.7795)	2.0100*** (3.5019)
Opening Up (OU)	0.3816* (1.5552)	0.5046** (2.0623)
Financial Development (FG)	-0.0738*** (-4.8445)	-0.0825 (-5.3288)
Environmental Regulation (ER)	-1.8308 (-1.3667)	-1.5012 (-1.1058)
Energy Structure (ES)	-0.2267*** (-6.9756)	-0.2393*** (-7.1901)
Urbanization Level(UL)	0.7416*** (13.0645)	0.7923*** (14.0328)
W*dep.var.	0.1550*** (3.8440)	
*spat*.*aut*.		0.1370*** (2.6234)
*R*−*squared*	0.7298	0.7216

Note: the values in () are T-test values, and *, * * and * * represent the significance levels of 10%, 5% and 1% respectively.

Industrial Structure has a considerable negative influence on the GDE at the 1% significance level, indicating that the larger the share of the secondary industry’s output value in GDP, the less conducive it is to improving the GDE. This demonstrates that China is still promoting industrialization quickly and that a large percentage of the country’s economy is still devoted to secondary industries, which has a detrimental effect on resource conservation and pollution abatement. The data also shows that China’s secondary industry output value accounted for 37.8% of GDP in 2020. Therefore, China’s industrial structure and that of developed nations still differ to some extent, and there is still room for future optimization and adjustment. As Zhu et al. [10] found, upgrading and rationalizing the industrial structure are both useful to raising the GDE.

Technological Progress and GDE have a very strong positive association, which is in line with the earlier prediction. The findings of Huang and Yu [57] also showed that technology research and development is a potent instrument for reducing the energy intensity of China. Ali et al. [58] also found that energy innovation has a positive impact on the environment. Therefore, the increase of RD expenditure is beneficial to the improvement of GDE. Taking 2020 as an example, the ratio of R&D value to GDP in Beijing is 0.0644, and its GDE is 1.1948. The ratio of R&D value to GDP in Ningxia is 0.0152, and its GDE is only 0.4617.

Opening Up has a positive effect on the GDE at the 1% significant level, which demonstrates that the more FDI a region attracts, the more favorable it is to improve the GDE. The outcome demonstrates that the “pollution heaven” hypothesis is not inapplicable in China, on the contrary, FDI plays a more “pollution halo” effect. With China paying increasing attention to environmental protection, the environmental protection threshold for FDI introduction is rising, as is the quality of FDI attracted. A significant quantity of high-quality foreign capital is brought in, which brings more advanced management experience and technology. It helps China’s economy grow while also reducing resource consumption and pollution emissions through spillover effect, which in turn improves the GDE. Some scholars confirmed this point. Peng [16] and Yu [24] all use cities in China as research objects and discover that FDI promotes green total factor production.

Financial Development has a negative impact on the GDE at a significant level of 1%, indicating that the higher the ratio of regional deposit and loan balance to GDP, the less conducive it is to improving GDE. This is different from the research results at the micro level. Ali et al., (2022) concluded that financial development is beneficial for improving company performance [59]. The possible reason is that China ’s present financial market development is not perfect, the bank credit structure is unreasonable. In particular, some banks attach importance to economic interests in the short term, and still flow a large amount of bank credit funds to high polluting industries. Yang and Ni [50] discovered that financial development reduces GDE in terms of financial scale and financial services, taking the Belt and Road countries including China as an example.

Environmental Regulation has a positive estimation coefficient, but its significance level test fails to pass. One plausible explanation is that environmental regulation "forced emission reduction" and "green paradox" exist at the same time, which offset each other, and eventually environmental regulations do not significantly affect GDE. Some scholars came to a similar conclusion. Wu et al. [60], Zhou and Li [61] all found that the GDE is impacted indirectly or uncertainly by environmental legislation.

As expected, Energy Structure and GDE have a significant negative association, which is similar to the research result of Wang et al. [27]. The outcome also demonstrates that coal’s position as China’s primary energy source has not changed. According to the report, China’s coal usage accounts for 56.8% of total energy consumption in 2020. In addition, the coal utilization in China is also extensive. A large amount of coal is directly used for combustion without desulfurization treatment, which produces a large number of pollutants.

The coefficient of Urbanization Level is positive, and it is 1% significant. It shows that the greater the proportion of urban resident population to the total population, the more it will promote the GDE. The similar finding was achieved by Li et al. [62]. They discovered that the improvement of urbanization level can promote the demographic structure transformation, thus promoting the improvement of GDE. In addition, the explanation for this conclusion is also strongly tied to the Chinese government’s new urbanization strategy. In comparison to traditional urbanization, modern urbanization prioritizes conservation, intensification, and ecological livability, places a premium on the development of ecological civilization, and actively promotes industrial ecology transformation.

## 5. Conclusion and recommendation

### 5.1 Conclusions

Promoting regional green development vigorously becomes an significant strategy to address China’s resource shortage and environmental degradation. Improving the GDE has become an important starting point to promote regional green development. A GDE evaluation index system is established in this paper, which includes four input indicators (labor, capital, energy and water resources), one desirable output (GDP) and three undesirable outputs (industrial wastewater discharge, industrial sulfur dioxide discharge and industrial smoke (powder) dust emissions). Then the temporal and spatial differentiation of GDE is discussed in this research. Based on this, this study creates a spatial econometric model to analyze the influencing factors of GDE. The outcomes are as follows:

1. The GDE varies significantly across provinces. During the sample period, only three regions (Beijing, Shanghai, and Tianjin) are at the forefront of GDE, while other provinces are not. The GDE of Guangdong and Fujian, for example, is not optimal, but it is relatively high. Green development in Gansu, Ningxia, Shanxi, Shaanxi, Sichuan, Chongqing, Guizhou, Hunan and Guangxi is inefficient and needs to be improved to a great extent in the future. The majority of provinces with higher GDE are situated in economically developed eastern coastal eastern regions, while the majority of provinces with lower GDE are located in economically underdeveloped interior areas.
2. During the sample period, the eastern, central, and western GDE shows a changing declining tendency. The GDE discrepancies between the three regions are also noticeable, with the eastern region having the greatest GDE, the western region having the second highest, and the central region having the lowest.
3. Except for 2020, all of the Global Moran’s I values of GDE are positive and significant, suggesting a considerable spatial correlation between provincial GDE. LISA map shows that the GDE shows the characteristic of "small agglomeration and large dispersion", mainly L-L agglomeration.
4. The spatial econometric model results reveal that Technological Progress (TP), Opening Up (OU) and Urbanization Level (UL) have significant positive impacts on GDE, while Industrial Structure (IS), Financial Development (FD) and Energy Structure (ES) have significant inhibitory effects on GDE, while Environmental Regulation (ER) has no significant effect.

### 5.2 Recommendations

The above research results show that GDE in most areas of China is still unsatisfactory. The level of regional green development should be further improved. Therefore, this paper offers pertinent policy recommendations from the three areas listed below.

1. The government should thoroughly account regional disparities when developing policies for green development. Because the green development in central and western China is relatively backward, the preferential tax and fee policies to support green development should be moderately inclined to the central and western China.
2. A cross-regional exchange and collaboration system for green development must be built for green growth. To compensate for the central and western regions’ lack of green development developed provinces in the eastern China should increase exchanges and cooperation with the backward central and western in technology, management, information, and other areas.
3. The relevant policies supporting green development still require improvement. The transformation of industrial structure must be accelerated, particularly relevant preferential policies for green industries, laws and regulations supporting green industries. Increased investments in science, technology, and innovation are required, as are the introduction of different innovation-focused preferential policies, the establishment of an enterprise innovation reward system, and tax cuts. The government should concentrate on vetting polluting businesses in order to increase the level of openness even more. when introducing foreign investment, raising the entry threshold, and implementing a strict environmental supervision system to actively optimize the foreign business environment. Green financial reform should be actively promoted, and the bank credit funds should be guided to flow more to green industries. Environmental regulatory policies must avoid the "one size fits all" phenomena, and the intensity of environmental regulation should be properly formulated based on the region’s development stage. The public should be encouraged by the government to get involved in the process of environmental oversight and information dissemination while enhancing the use of market means such as environmental taxes and tradable pollution discharge permits. In addition, it is necessary to actively plan new energy industries, optimize the energy grid, boost the share of clean energy applications like hydrogen, solar, and wind energy, and reduce the fossil energy consumption. The new urbanization strategy should be actively promoted, and urbanization expansion should be kept within an acceptable range to ensure maximum benefits and minimum pollution output. To ensure the balance of material flow and energy flow in cities and towns, a system for ecological balance needs to be established.
